# Direct and Indirect Costs Related to Physical Activity Levels in Patients with Diabetes Mellitus in Spain: A Cross-Sectional Study

**DOI:** 10.3390/healthcare10040752

**Published:** 2022-04-18

**Authors:** Antonio Sarria-Santamera, Zhanna Alexeyeva, Mei Yen Chan, Miguel A. Ortega, Angel Asunsolo-del-Barco, Carlos Navarro-García

**Affiliations:** 1 Department of Medicine, Nazarbayev University School of Medicine, Nur-Sultan 010000, Kazakhstan; zhanna.alexeyeva@nu.edu.kz (Z.A.); yen.chan@nu.edu.kz (M.Y.C.); 2 Department of Medicine and Medical Specialities, Faculty of Medicine and Health Sciences, University of Alcalá, 28801 Alcala de Henares, Spain; miguel.angel.ortega92@gmail.com; 3 Ramón y Cajal Institute of Sanitary Research (IRYCIS), 28034 Madrid, Spain; angel.asunsolo@uah.es; 4 Department of Surgery, Medical and Social Sciences, Faculty of Medicine and Health Sciences, University of Alcalá, 28801 Alcala de Henares, Spain; 5 Faculty of Health and Sports Sciences, Universidad Alfonso X, Villanueva de la Cañada, 28691 Madrid, Spain; navarrog@uax.es

**Keywords:** diabetes mellitus (DM), physical activity, healthcare costs, sedentary lifestyle

## Abstract

Diabetes mellitus (DM) is a global public health concern. DM is importantly linked to the modern lifestyle. Lifestyle-based interventions currently represent a critical preventive and therapeutic approach for patients with DM. Increasing physical activity has proven multiple benefits to prevent this condition; however, there is still room for further progress in this field, especially in terms of the effect of exercise in patients with already established DM. This study intends to examine the economic relationship between physical activity and direct/indirect costs in patients with DM. We analyze a national representative sample (*n* = 1496) of the general population of Spain, using available data from the National Health Survey of 2017 (NHS 2017). Our results show that 63.7% of the sample engaged in some degree of physical activity, being more frequent in men (67.5%), younger individuals (80.0%), and those with higher educational levels (69.7%). Conversely, lower levels of physical activity were associated with female sex, older subjects, and various comorbidities. Our study estimates that 2151 € per (51% in direct costs) patient may be saved if a minimum level of physical activity is implemented, primarily, due to a decrease in indirect costs (absenteeism and presenteeism). This study shows that physical activity will bring notable savings in terms of direct and indirect costs in patients with DM, particularly in some vulnerable groups.

## 1. Introduction

Diabetes mellitus (DM) is a global public health concern characterized by elevated plasma glucose (hyperglycemia) due to failures in insulin production, action, or a mixture of both [1]. The development and evolution of DM result from the interplay between different genetic and environmental factors. An unhealthy lifestyle appears to be a major contributor to multiple mechanisms implicated either in the causation as well as the development of its complications [2,3,4]. The growing global tendency of obesity, physical inactivity, and energy-dense diets worldwide will be leading to an unprecedented rise in the number of patients with DM [5]. Indeed, the International Diabetes Federation projects that 693 million people will have DM by 2045 in the world [6,7].

DM represents a considerable burden for patients, healthcare systems, and society, both in terms of the direct costs related to medical care as well as indirect costs because of the diminished productivity tied to DM-related morbidity and mortality [8]. In 2017, the global expenditure on DM patients aged 18–99 years reached USD 850 billion, and it is expected to rocket up to USD 958 billion by 2045 [6]. In Spain, the costs of DM have been estimated to represent 8% of the national healthcare expenditure [8], and the prevalence of self-reported DM is estimated to be between 8 and 14% with an incidence of 11.6 new cases per 1000 persons/year [9,10,11,12,13,14]. Thus, there is evidence of the urgent need for finding adequate and economic measures to limit the huge burden of DM in different countries such as Spain.

Lifestyle-based interventions represent a great alternative both for the prevention and clinical management of DM [15,16]. Adopting and maintaining physical activity is critical for blood glucose management and overall health in individuals with DM [17]. Exercise improves blood glucose control, reduces cardiovascular risk factors, contributes to weight loss, and improves well-being [18,19]. The maintenance of normal blood glucose depends largely on the coordination and integration of the sympathetic nervous and endocrine systems. Contracting muscles increase the uptake of blood glucose. The intensity and duration of physical activity are critical factors to influence exercise fuel use and improve insulin action assisting with the management of glucose levels.

All individuals, not only persons with DM, should be recommended to engage in tailored physical activity and exercise, adapted to their personal needs, using appropriate behavior-change strategies to promote the adoption and maintenance of lifetime physical activity [20]. However, sedentary behavior—waking behaviors with low energy expenditure—is a ubiquitous and significant population-wide influence on cardiometabolic health [21,22] and is associated with increased mortality and morbidity [23]. The Spanish population shows an overall low prevalence of physical activity: 27% of adults and 55.4% of children and adolescents do not meet international physical activity recommendations. Approximately more than 40% of old adults are sedentary [24].

The cost-effectiveness of behavioral interventions to prevent DM has been consistently reported, but there is limited knowledge of the economic benefit in patients with already established DM [25]. Exercise referral schemes have been found to result in a small improvement in the number of people who increase their levels of physical activity with ICER (incremental cost-effectiveness ratio) for those interventions compared to usual care to be around £76,000 per QALY (quality-adjusted life-year) [26]. Although robust evidence on the significant costs associated with DM in the Spanish population can be found in previous studies [9], there are little data either on the prevalence of physical activity in this group of patients or on what is the impact of sedentarism on the associated costs. Therefore, this work aims to describe the physical activity level in the Spanish DM population and to compare direct and indirect costs in patients with DM performing physical activity versus those with a sedentary lifestyle.

## 2. Materials and Methods

This is a cross-sectional study of DM in Spain from a societal perspective. This work will estimate direct and indirect costs, using the National Health Survey of 2017 (NHS 2017), which is a national representative sample of the Spanish no-institutionalized general population (*n* = 23,089) over 14 years of age. Trained interviewers conducted face-to-face interviews and obtained data on socio-economic characteristics, health and functional status, lifestyle and health behaviors, comorbidities, and health services utilization. For this work, we selected participants in the NHS 2017 who provided an affirmative response to the following questions: Have you had diabetes in the last 12 months? Has your doctor told you that you have diabetes? For at least the last 6 months, to what extent have you been limited due to a health problem in doing the activities that people usually do? Do you have difficulty walking 500 m on level ground without any kind of walking aid? Do you have difficulty going up or down 12 steps?

The questionnaire included the following question to investigate the physical activity of participants: “Which of these options best describes the frequency with which you do some physical activity in your free time?”, providing 4 possible answers: “(1) I don’t exercise. I spend my free time almost completely sedentary (2) I do some physical activity or occasional sports. (3) I do physical activity several times a month (4) I do sports or physical training several times a week”. For this analysis, two categories were created: those who do not have any physical activity (response 2), and all those with at least minimum physical activity, combining the 3 groups for those who indicated to engage in physical activity (responses 2–4).

### 2.1. Direct Costs

The variables included for the calculation direct costs were: number of visits to general practitioner or family doctor (GP) in the last 2 weeks; number of visits to specialist doctors in the last 2 weeks; number of visits to the emergency room; and number of days being hospitalized in the last 12 months.

We estimated the unitary costs of the variables related to the utilization of health services using the average of the available tariffs calculated by the different Spanish regional Healthcare Services for each specific service [27,28,29,30,31,32,33]. For costs of visits to general practitioners and specialist doctors, and given the usual chronic nature DM, tariffs of successive consultations were used (not of first consultations).

The following unitary costs, updated to euros in the year 2020, were used in this work: Visit to emergency service €149.84; Visit to general practitioner of family doctor visit €41.80; Visit to specialist doctor 87.82 €; Hospital-day 483.69 €; Cost of working day 75.07 €.

### 2.2. Indirect Costs

Indirect costs were estimated using two variables related to the loss of productivity: absenteeism and presenteeism. We assumed that loss of productivity associated with absenteeism corresponds to “days in bed”. Absenteeism is calculated from the number of days spent in bed (more than half of the day) in the last two weeks. Presenteeism reflects the situation that although the subject is present in his/her job, they are not fully productive. In this work, it was estimated by the number of days during the last two weeks suffering a restriction in their activities of daily. According to other studies, occupational presenteeism, when it occurs, may represent a 30% loss of productivity [34], so this value will be used for calculating productivity losses related with presenteeism.

Information on salaries was obtained from the Survey of Salary Structure elaborated by the National Institute of Statistics of the year 2016. To calculate the monetary value of one day of productive work, the annual salary was divided by 250. This figure was multiplied by the days lost of work to obtain the cost of absenteeism and multiplied by 0.30 to calculate the cost of days of labor lost by presenteeism. For determining individual salaries, the regional salary average was used (Table 1).

### 2.3. Statistical Analysis

A descriptive analysis of the data was carried out. Qualitative variables were described with their frequency distribution and the quantitative variables with the mean and the standard deviation. Pearson χ^2^ will be used to identify statistical differences in these analyses for categorical variables and ANOVA test in the case of continuous variables.

We conducted binomial negative multivariable regressions to identify the independent effect of physical activity on the set variables used for calculating direct and indirect costs, adjusting for relevant confounding factors: age, sex, marital status, educational level, and presence of other relevant chronic long-term diseases. We tested all the models conduced for overdispersion, using the residual deviance, not finding it in any of them.

Using the Odds Ratios that reflected the independent effect that physical activity explains in each of those negative binomial multivariable models, we calculated the proportion that may be attributable to physical activity. All the costs were estimated on a 12-month basis. The overall incremental savings of each of the items valued was obtained by multiplying the unit cost of each of the services/days of productivity losses by the number of days of absenteeism or presenteeism or by the number of units of medical care reported by each participant. Negative binomial regression models will be used, as they overcome the over-dispersion of Poisson regression.

*p* values lower than 0.05 will be considered to represent such extreme differences that would be very unlikely to occur by chance alone and would therefore be estimated as statistically significant. Statistical analysis will be obtained on R programming.

## 3. Results

### 3.1. Socio-Demographic and Clinical Characteristics of the Patients

Data from the 1496 respondents to the questionnaire that met the inclusion criteria were analyzed providing representative data of the Spanish DM population. Table 1 presents the socio-demographic and clinical characteristics of respondents. It illustrates the different levels of physical activity reported by the participants. Overall, 63.7% of the sample engaged in some degree of physical activity. Physical activity was more frequent in men, younger age groups, and those with higher education attainment. As shown in Table 1, acute myocardial infarction, stroke, chronic kidney disease, depression, anxiety and obesity or overweight were comorbidities associated with lower physical activity.

### 3.2. Direct and Indirect Costs Related to Physical Activity Levels

Table 2 displays the differences in use of services and reduction in functional capacity by those with and without physical activity. Apart for visits to specialist doctors, persons with DM engaging in physical activity had a significantly lower utilization of services and losses of daily productivity.

The OR of the negative binomial multivariable regression analysis results is shown in Table 3 indicating the independent effect of physical activity on each of the dependent variables. Except for visits to general practitioners and specialists, the ORs were statistically significant, indicating that physical activity in DM patients was an independent factor having a significant effect in a lower use of healthcare resources and in productivity losses. No cost estimations have been obtained and included in the analysis therefore for visits to GP or specialists. Table 3 also reflects the attributable effect that physical activity may have on decreasing health services utilization and the number of days of restriction of activities estimated in an annual base. Without including savings associated with visits to GP and specialists whose multivariable models did not find a statistical association, an estimated 21,514.95 € per patient may be saved, being 51% reductions in direct medical costs (hospital-days and emergency visits) and 49% in savings that may be attributed to a decrease in days lost because of restrictions of activities. As the population included in this analysis may represent 6% of the population over 15 years old in Spain, it may be estimated that improving physical activity in the Spanish DM population may be associated with overall savings in direct and indirect costs that may represent 35% of total Spanish healthcare expenditures.

## 4. Discussion

The main finding of this work is that patients with DM who report even minimal physical activity show a lower use of healthcare services and suffer fewer days of restriction of their activity. The monetary values of those effects represent that if the Spanish DM population had at least that minimal physical activity, annual savings of 2151.95 € per patient would be achieved, being 51% of them related to higher use of services observed in patients with a sedentary lifestyle and 49% associated with a lower functional status related with a reduced restriction of productivity. This work also confirms the low proportion of DM patients in Spain with an active lifestyle and the existence of significant differences in physical activity across socio-demographic groups. Women, older individuals and patients with low educational levels are less physically active. These results reflect the need to target those population groups for improving their physical activity. Likewise, a direct association between sedentarism, anxiety, and depression is observed in our sample. Previous studies have reported that both mental disorders are two major contributors to the excess costs in DM patients [35]. Therefore, increasing physical activity levels that could aid in the management of depression and anxiety may have a profound impact from an individual, healthcare, and socio-economic perspective.

The low proportion of patients with DM engaging in physical activity may be considered a major public health concern. Physical activity has been linked to significant favorable changes in metabolic dimensions associated with better control and outcomes in DM. Being physically active enhances the body’s sensitivity to insulin while countering insulin resistance, with added benefits for people with DM [36]. Sedentary behaviors, prolonged sitting or reclining while awake, including television viewing, working on a computer, and driving, are related to adverse health outcomes [37]. In this sense, previous studies have evidenced positive associations between the amount of sitting and the risk of complications and premature mortality in people with DM [38,39,40]. The design and characteristics of this study prevent determining the concrete mechanisms involved in the link between physical activity with reduced direct medical and indirect social costs. However, a plausible explanation based on previous works is that DM patients engaging in physical activity may have better glycemic and HbA1c values as well as in other metabolic pathways [41], leading to an overall improvement in cardiovascular risk factors [42]. Exercise improves whole-body metabolic health, leading to an increase in glycolipid uptake and utilization, improved insulin sensitivity, optimized body mass index, and modulated DNA methylation. Some cytokines such as irisin, osteocalcin and adiponectin are closely related to exercise and metabolic diseases [43]. Nevertheless, further research exploring the biological mechanisms underlying the benefits of physical activity in DM is necessary, providing a theoretical basis for exercise therapy in these individuals.

Patients with DM may have limited information about the consequences of this disease. As DM is an asymptomatic silent condition, except for acute or chronic complications, it is complex for patients to perceive it as a serious disease. A strong patient–professional relationship built in a context of mutual trust, considering patients’ values and perspectives, providing clear and adapted information, and supporting them to introduce physical activity in their daily lives and existential circumstances, is critical to strengthening self-management [44]. It is frequent that patients with DM overestimate their daily physical activity, underestimate the benefits of practicing exercise, or have multiple difficulties in terms of adherence [45,46,47]. Healthcare workers should initiate discussions with DM patients regarding physical activity, communicating practical applications on how to have a more active lifestyle [48]. Every clinically stable patient (including the frailest) can benefit from exercise tailored to their circumstances [49]. Exercise is a safe and effective treatment modality to assist in the control of glucose levels and reduce complications for individuals with DM. As every patient is different, a single solution tailored purely to the primary morbidity is unlikely to work, keeping in mind that any increase in the level of physical activity, however small, is likely to be beneficial. Findings from a growing number of studies suggest that healthcare systems should consider the possibility for reimbursement of physical activity programs, given the health benefits of physical exercise for the management of patients with DM, and more broadly for improving the health of the general adult and elder population [50]. Recently, the treatment of DM and other chronic diseases is turning to a more holistic approach. Specifically, social prescription is a new concept of referring patients to a wide range of non-clinical services, such as arts on prescription, exercise referral, etc. [51]. Considering the clinical characteristics of individuals with type 2 DM, with a high proportion of women and elderly, interventions aimed at increasing muscle strength, balance and blood parameters are fundamental in improving the functionality of these individuals. Whole-body vibration has also been a useful strategy in the management of the symptoms and disabilities associated with DM [52].

This study has certain limitations. The survey did not focus on physical activity and offers a limited perspective on its description and quantification. For defining physical activity, other studies have used detailed and specific questionnaires, while here, we rely on a single question. The cross-sectional design of the NHS2017 limits inferring causality relationships as well as estimating the implications of reducing healthcare utilization or days of restricted activities in the long-term costs of DM. Data on DM are self-reported, and no information on clinical characteristics, duration, or progress of DM is available, although multivariable models were adjusted by variables that have a relevant impact on DM, such as age, sex, and comorbidities.

The costs for health services have been calculated using existing and available data of prices for healthcare from several Spanish regions, although not all of them correspond to same year, using a methodology reported elsewhere [52]. We only considered work absenteeism and presenteeism for indirect costs. As the NHS 2017 does not include specific direct questions for absenteeism and presenteeism, we had to equate those dimensions with the questions available in the survey. Salaries were estimated based on regional data from official national statistics. Indirect costs related to loss of productivity have been calculated using the human capital approach, which may produce higher estimates than using the friction cost approach [53,54]. 

Our cost estimates, however, may also yield conservative estimates because the data we analyzed did not allow us to include the direct costs attributable to other medical resources whose use may be also higher in the case of DM (medicines, diagnostic tests). Finally, this work is not providing an economic of the impacts either on health outcomes (acute or chronic complications related to DM) or utilities (in the form of QALYs gained) [50]. Additional information regarding the potential benefits and costs of structured exercise is needed, including data from rigorously conducted clinical trials to provide information concerning the efficacy and cost-effectiveness of such programs. This issue is critical because there is a strong association between the availability of spaces for physical activity and the levels at which it is performed. Apart from encouraging physical activity, it is necessary to implement programs for patients with DM to maximize the benefits of exercise.

The strength of this study is the use of a national representative sample of people with diabetes that has demonstrated validity to investigate health status, determinants of health, and utilization of health services at the population level. The National Health Survey is a significant source of information to monitor the health of the Spanish population through the collection and analysis of data on a broad range of health topics. A major strength lies in the ability to categorize these health characteristics by relevant demographic and socio-economic characteristics, allowing monitoring trends in illness and disability and tracking progress toward achieving national health objectives. These data are also used by the public health research community for epidemiologic and policy analysis of such timely issues as characterizing those with various health problems, determining barriers to accessing and using appropriate healthcare and evaluating the impact of health programs.

## 5. Conclusions

Low levels of physical activity are associated with a significant impact on an excess utilization of healthcare services and deteriorated functional status leading to restriction of activities and losses in productivity. A sedentary lifestyle among patients with DM represents a massive socio-economic burden. Consistent with previous studies, our findings support that physical activity and exercise provide strong evidence for public policymakers to consider structured exercise and physical activity programs worthy of insurance reimbursement for patients with DM. Public health authorities should consider the implementation of effective practices that have shown promising results in increasing physical activity [55,56]. The goal is to perform at least 150 min per week of moderate-intensity physical activity. One way to do this is to try to fit in at least 20 to 25 min of activity every day. In addition, on 2 or more days a week, include activities that work all major muscle groups (legs, hips, back, abdomen, chest, shoulders, and arms) [57].

## Figures and Tables

**Table 1:** Socio-demographic and clinical characteristics of the sample analyzed.

Variables		%	Physical Activity (%)	*p*
Total number of cases included		1496	63.7	
Sex	Male	56.1	67.5	
	Female	43.9	58.8	0.001
Age (Mean = 69.88 SD = 12.56)	15–30	0.3	80.0	
	31–45	5.1	64.5	
	46–65	34.1	61.8	
	66–74	32.0	70.5	
	>74	28.5	58.1	0.002
Marital status	Single	11.5	68.6	
	Married	60.7	64.6	
	Widowed, separated, divorced	27.7	59.7	0.080
Education	Less than primary	22.5	56.3	
	Primary	32.0	61.8	
	Secondary	36.7	68.5	
	University	8.8	69.7	0.001
Body Mass Index	Normal weight	22.0	68.0	
	Overweight-Obesity	72.5	63.5	0.251
Hypertension		58.7	63.1	0.562
Hyperlipidemia		51.0	63.2	0.663
Myocardial infarction or Stroke		3.1	47.8	0.023
Chronic kidney disease		6.9	49.5	0.002
Anxiety		7.6	55.3	0.051
Depression		10.0	53.0	0.004
Levels of physical activity	No	36.3		
	Some	63.7		
Visits to a general practitioner or family doctor in the last four weeks	0	50.7	67.7	
	1	37.8	60.2	
	2 or more	11.5	58.8	0.031
Visits to a specialist doctor in the last four weeks ≥1		22.2	56.9	0.036
Hospitalizations in the last 12 months ≥1		13.5	55.4	0.009
Visits to emergency services in the last 12 months ≥1		38.8	56.0	0.000
Spending at least half of the day in bed in the last 2 weeks		4.1	45.2	0.000
Suffering a restriction in activities of daily life during the last 2 weeks		12.7	51.1	0.000

**Table 2:** Mean values of use of services and days of loss of productivity losses in diabetic persons.

	Sedentary	Physical Activity	*p*
Visits to General Practitioners or family doctors	0.68	0.56	0.003
Visits to specialist doctors	0.31	0.27	0.315
Number of hospital days	3.13	1.58	0.042
Visits to emergency services	0.68	0.42	<0.000
Days suffering a restriction in activities of daily life	1.35	0.74	<0.000
Days spent at least half of the day in bed	0.37	0.12	<0.000

**Table 3:** Attributable savings to physical activity per patients: OR for adjusted binomial multivariable regression models of the independent effect of physical activity in the dependent variables.

Variables	OR	*p*-Value	Attributable Risk (%)	Observed per Person	Expected per Person with Reductions Attributable to Physical Activity	Expected Savings per Person Attributable to Physical Activity	Expected Savings per Person Attributable to Physical Activity in 12 Months
Direct costs: Medical care							
Visits to general practitioner or family doctor	0.73	0.065	−0.14	0.96	--	--	--
Visits to specialist doctor	0.69	0.368	−0.11	0.36	--	--	--
Days of hospitalization	0.58	0.011	−0.73	3.07	0.84	719.30	1078.55
Visits to emergency services	0.84	0.001	−0.19	0.75	0.61	38.67	20.82
							1099.37
Indirect costs: Productivity losses							
Days suffering a restriction in activities of daily life	0.58	0.000	−0.48	0.96	0.05	34.37	652.60
Days spent at least half of the day in bed	0.37	0.000	−0.98	0.21	0.01	15.38	399.98
							1052.58
						Total	2151.95

## Data Availability

The datasets used and/or analyzed during the present study are available from the corresponding author on reasonable request.
